# NK Cells in Protection from HIV Infection

**DOI:** 10.3390/v14061143

**Published:** 2022-05-25

**Authors:** Nicole F. Bernard, Khlood Alsulami, Erik Pavey, Franck P. Dupuy

**Affiliations:** 1 Research Institute of the McGill University Health Centre (RI-MUHC), Montreal, QC H4A3J1, Canada; khlood.alsulami@mail.mcgill.ca (K.A.); paveyerik@gmail.com (E.P.); dura007@hotmail.fr (F.P.D.); 2 Division of Experimental Medicine, McGill University, Montreal, QC H4A 3J1, Canada; 3 Infectious Diseases, Immunology and Global Health Program, Research Institute of the McGill University Health Centre, Montreal, QC H4A 3J1, Canada; 4 Division of Clinical Immunology, McGill University Health Centre, Montreal, QC H4A 3J1, Canada

**Keywords:** natural killer cells, HIV, HIV-exposed seronegative, HLA, HLA-B*57, killer immunoglobulin-like receptors

## Abstract

Some people, known as HIV-exposed seronegative (HESN) individuals, remain uninfected despite high levels of exposure to HIV. Understanding the mechanisms underlying their apparent resistance to HIV infection may inform strategies designed to protect against HIV infection. Natural Killer (NK) cells are innate immune cells whose activation state depends on the integration of activating and inhibitory signals arising from cell surface receptors interacting with their ligands on neighboring cells. Inhibitory NK cell receptors use a subset of major histocompatibility (MHC) class I antigens as ligands. This interaction educates NK cells, priming them to respond to cells with reduced MHC class I antigen expression levels as occurs on HIV-infected cells. NK cells can interact with both autologous HIV-infected cells and allogeneic cells bearing MHC antigens seen as non self by educated NK cells. NK cells are rapidly activated upon interacting with HIV-infected or allogenic cells to elicit anti-viral activity that blocks HIV spread to new target cells, suppresses HIV replication, and kills HIV-infected cells before HIV reservoirs can be seeded and infection can be established. In this manuscript, we will review the epidemiological and functional evidence for a role for NK cells in protection from HIV infection.

## 1. Introduction

Some people remain HIV seronegative despite high levels of exposure to this virus. They are referred to as HIV-exposed seronegative (HESN) individuals [1]. Studying HESNs may provide information that enhances our understanding of the mechanisms that underlie their apparent resistance to HIV infection. This information has the potential to guide the development of novel prophylactic HIV therapies.

Several groups have screened HESNs for the presence of adaptive HIV-specific T cells and antibody responses. Such responses have been found in many but not all cohorts of HESN subjects [2,3,4,5,6,7]. HIV-specific T cell responses in HESN were often of a low breadth and magnitude, and their presence did not always predict maintenance of seronegative status [8,9,10,11,12]. The STEP HIV vaccine trial, designed to induce strong T cells responses, failed to protect against HIV infection [13,14]. Furthermore, HIV-specific memory responses, even if present in HESNs, would be expected to take days following an exposure to HIV to develop into effector responses able to kill HIV-infected cells and prevent the establishment of HIV infection. In an animal model for HIV infection in humans, i.e., rhesus macaques infected intrarectally with simian immunodeficiency virus, starting antiretroviral therapy (ART) as early as day 3 post infection failed to prevent viral load rebound when ART was stopped 24 weeks later [15]. This observation is consistent with viral reservoirs refractory to eradication being seeded early during the eclipse phase of HIV infection before virions and infected cells are detectable in the circulation [16]. Together, these observations suggest that adaptive HIV-specific immune responses induced by exposure to HIV are likely to be generated too late to prevent the seeding of viral reservoirs and establishment of HIV infection resulting from exposure to HIV [15,16]. Thus, adaptive immune responses detected in HESN more likely identify individuals as having been previously exposed to the virus rather than protect from subsequent HIV exposures [17].

Innate Natural Killer (NK) cell immune responses with anti-HIV activity are induced more rapidly than adaptive immune responses [18]. NK cells, as components of the innate immune response, have the potential to protect against HIV infection. NK cells play an important role in anti-viral and anti-tumor activity [19,20]. They are primed to respond rapidly to HIV-infected cells, which have increased expression of ligands for germline-encoded activating NK receptors (NKRs) as occurs on HIV-infected cells [21]. HIV-infected cells downmodulate their cell surface expression of major histocompatibility complex (MHC) class I antigens [22,23,24,25]. Upon activation, NK cells secrete cytokines with anti-viral activity and chemokines that block the CCR5 co-receptor for HIV entry into new target cells and degranulate, which can lead to the lysis of infected cells [19,26,27,28]. Whether NK cells respond to stimuli or remain resting depends on the integration of signals received from inhibitory and activating NKRs [29]. Signals from inhibitory NKRs tend to dominate those from activating NKRs [29,30,31]. NK cells can be activated directly when the integration of signals from inhibitory and activating NKRs upon binding their ligands on neighboring cells favors activation.

In this review, we will focus on how KIR/HLA combinations that contribute to NK cell education influence NK cell responses to prevent HIV infection.

## 2. Basis of NK Cell Education

During their development, NK cells are educated through the interaction of their germline encoded inhibitory NKRs with self MHC class I (or human leukocyte antigen (HLA)) ligands expressed on adjoining cells [32,33,34,35]. A consequence of NK cell education is tolerance to self-cells as the interaction of inhibitory NKRs with their HLA ligands transduces inhibitory signals that suppress NK cell activation [36,37]. However, education also primes NK cells to respond rapidly to aberrant cells with reduced levels of HLA ligands due to virus infection, tumor transformation, or cell damage [35,38]. HIV infection downmodulates cell surface MHC expression through the action of HIV negative regulatory factor (Nef) and viral protein U (Vpu), which are HIV-encoded accessory proteins with multiple functions [22,23,24,25].

HLA-E interacts with the inhibitory NKR NKG2A to educate NK cells [39]. Many HLA allotypes are ligands for inhibitory killer immunoglobulin-like receptors (KIRs). KIRs are a large family of receptors that include both activating and inhibitory counterparts expressed on NK cells and other lymphocyte subsets [40]. Binding of inhibitory KIRs to their HLA ligands transduces intracellular inhibitory signals that support tolerance to healthy HLA-expressing autologous cells [29,30,31]. The *KIR* and *HLA* regions are polygenic and characterized by a high degree of polymorphism. They are encoded on chromosomes that segregate independently from each other allowing for a great diversity of receptor ligand combinations. [41,42,43]. NKRs, including KIRs, are expressed stochastically on overlapping NK cell subsets. Thus, both educated and uneducated NK cells can co-exist as can NK cells with varied levels of education and responses to stimulation, due to allelic *KIR* and *HLA* variation [35,44,45]. The inhibitory KIR^+^ NK cell subsets unable to interact with self HLA during development remain uneducated and are hyporesponsive unless activating signals predominate [32,34,46,47]. The depth of NK cell education, which depends on the number of inhibitory NKR-ligand interactions, inhibitory KIR/HLA ligand density and the affinity of the interactions between the two during the education process, correlates with the level of activation mature NK cells achieve when they encounter cells with aberrant HLA expression levels [33,35,38].

## 3. KIR NK Receptors

The genetic *KIR* region is located within human chromosome 19q13.4. Information generated using full haplotype multiple sequence alignment had identified 14 loci and 1 intergenic locus containing 9 genes in this region [48]. KIR nomenclature reflects their protein structure [49]. After “KIR” is the number of extracellular immunoglobulin-like domains (2D or 3D), followed (S or L) for short or long intracellular domains characteristic of activating or inhibitory receptors, respectively. P refers to pseudogenes in the KIR nomenclature. A number follows, which indicates the sequence in which each allele or protein was named [50]. The long cytoplasmic domains of inhibitory receptors have an immunoreceptor tyrosine-based inhibitory motif that recruits SRC homology 2 (SH2) domain containing protein tyrosine phosphatases such as SHP1 and SHP2 [30]. The short cytoplasmic domain of activating receptors associates with adaptor molecules with immunoreceptor tyrosine-based activating motifs that recruit protein tyrosine kinases [29].

Among the inhibitory KIRs, the ligands for KIR3DL1 are a subset of HLA-A and -B antigens having an HLA-Bw4 motif, defined by amino acids present at positions 77–83 of the HLA heavy chain (Table 1) [51,52]. A dimorphism at position 80 of the Bw4 heavy chain divides these isotypes into those with an isoleucine (*80I) or threonine (*80T) at this position, which influences HLA cell surface expression and binding affinity to KIR3DL1 [35,45,53]. HLA-Bw6 allotypes do not interact with KIR3DL1 and NK cells from *HLA*-*Bw6* homozygotes expressing KIR3DL1, as their only inhibitory KIR remain uneducated [32]. KIR3DL1 allotypes can be categorized as KIR3DL1-null with no detectable cell surface expression, KIR3DL1-low and KIR3DL1-high allotype groups based on their cell surface expression levels [35,54]. In general, KIR3DL1-high receptors have a higher affinity for Bw4*80I than Bw4*80T allotypes while the avidity of KIR3DL1-low receptors to these two HLA-Bw4 groups is similar [35]. The inhibitory KIRs KIR2DL1, KIR2DL2, and KIR2DL3 recognize HLA-C allotypes, which can be dichotomized into C1 and C2 groups. C1 allotypes have an asparagine at position 80 of their heavy chain and are ligands for KIR2DL3. C2 group allotypes have a lysine at this position and are ligands for inhibitory KIR2DL1 and activating KIR2DS1 receptors [44,55,56]. KIR2DL2 is an intermediate receptor, which also binds C1 allotypes and some C2 allotypes (Table 1) [44,55,57].

**Figure 1:**
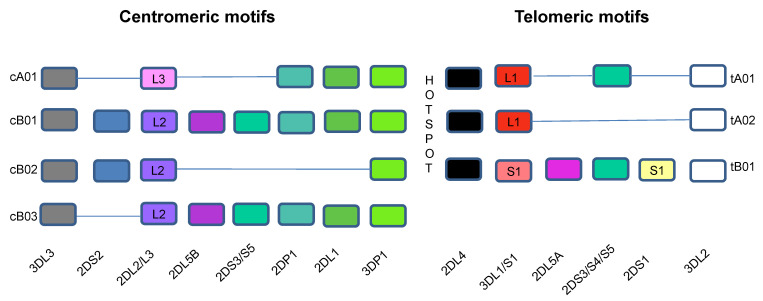
Killer Immunoglobulin-like Receptor Haplotypes. *KIR* genes are organized into haplotypes A and B. *KIR* genes shown in the bottom row are designated without “KIR” before their names. The different colors are used to identify different genes. Haplotype A is comprised of four framework genes present in most *KIR* haplotypes (*KIR3DL3* at the centromeric end, *KIR3DL2* at the telomeric end and the pseudogene *KIR3DP1* and *KIR2DL4* in the middle) plus genes encoding inhibitory KIRs *KIR2DL1*, *KIR2DL3*, *KIR3DL1*, activating KIR *KIR2DS4* and pseudogene *KIR2DP1*. The more diverse group B haplotypes include the framework genes with various combinations of genes encoding inhibitory KIRs *KIR2DL2* and *KIR2DL5A/B* and activating KIRs *KIR2DS1*, *KIR2DS2*, *KIR2DS3*, *KIR2DS5,* and *KIR3DS1*. More than 90% of the 68 haplotypes that have been sequenced to date using full haplotype multiple-sequence alignments are composed of one of four centromeric and one of three telomeric *KIR* motifs that include combinations of *KIR* genes in linkage disequilibrium with each other, which are usually inherited together. The intergenic region between *KIR3DP1* and *KIR2DL4* is a hotspot between the centromeric and telomeric regions, which allows for frequent recombination between the two regions. The centromeric region is delimited by the framework genes *KIR3DL3* and *KIR3DP1,* while the telomeric region is delimited by framework genes *KIR2DL4* and *KIR3DL2. KIR2DP1* and *KIR2DP1* are pseudogenes. *cA01* = genes in the centromeric region of *KIR* haplotype A 1; *cB01* = genes in the centromeric region of *KIR* haplotype B 1; *cB02* = genes in the centromeric region of *KIR* haplotype B 2; *cB03* = genes in the centromeric region of *KIR* haplotype B 3; *tA01* = genes in the telomeric region of *KIR* haplotype A 1; *tA02* = genes in the telomeric region of *KIR* haplotype A 2; *tB01* = genes in the telomeric region of *KIR* haplotype B 1.

Historically viewed, the human *KIR* system segregates into two groups of haplotypes (*A* and *B*), which are distinguished, at least in part, by their activating *KIR* gene content (Figure 1) [42,58,59,60,61]. The group *A* haplotypes are dominated by genes encoding inhibitory receptors [60]. The exception to this is the *KIR2DS4* locus, which encodes an activating receptor that is often truncated and not cell surface expressed [62,63]. The group *B* haplotypes encode more variable and greater numbers of genes encoding inhibitory receptors and varying numbers activating receptors [60]. *KIR* haplotypes share three framework genes and one pseudogene. *KIR3DL2* and *KIR2DL3* are located at the centromeric and telomeric ends of the *KIR* region while the pseudogene *KIR3DP1* and *KIR2DL4* are in the middle, together framing two regions of variability of the *KIR* locus [64]. A 14 kb intergenic region separates *KIR2DP1* and *KIR2DL4* and divides the *KIR* locus into centromeric and telomeric regions [40]. This intergenic region is a site of reciprocal recombination, which allows the centromeric and telomeric regions to reassort into combinations that form new variant *KIR* haplotypes [58,65]. The genes in the telomeric and centromeric regions are in linkage disequilibrium with each other and tend to be inherited together [59,61]. Full haplotype DNA multiple sequence alignments are now available for 68 *KIR* haplotypes [48]. This information has refined and confirmed the sequence alignments of several *KIR* haplotypes [48,59,61]. Of the 13 *KIR* haplotypes identified by full *KIR* region sequencing, 6 were found in only one person [48]. More than 90% of the 68 fully sequenced *KIR* haplotypes are composed of one of four centromeric and one of three telomeric motifs (Figure 1). The less frequent *KIR* haplotypes include gene duplications, deletions, insertions, and hybridizations of *KIR* genes present in the more frequent haplotypes [59,61].

## 4. Epidemiological Studies Supporting a Role for NK Cells in Protection from HIV Infection

One of the first publications implicating NK cells in protection from HIV infection showed, in a cohort of highly HIV exposed Vietnamese injection drug users (IDUs), that IDU HESN persons had enhanced NK cell responses to HLA null cells compared to individuals at low risk for HIV infection and IDU persons living with HIV (PLWH) [66]. The elevated NK cell activity in IDU HESN individuals was higher than that in IDU PLWH for NK cells isolated from IDU PLWH at time points both before and after seroconversion [66]. The observation that lower levels of NK cell activity in IDU PLWH predated HIV seroconversion suggested that NK cell-mediated protection from HIV infection was genetically determined.

Epidemiological studies conducted on samples from longitudinally followed PLWH enrolled in the Multicenter AIDS Cohort Study found an association between certain KIR3DL1/HLA and KIR3DS1/HLA combinations with time to the acquired immunodeficiency syndrome (AIDS) (using the 1987 US Centers for Disease Control and Prevention’s definition for AIDS), time to CD4 counts of <200 cells/mm^3^ and HIV viral load control [67,68]. In these studies, *Bw6* homozygotes with uneducated KIR3DL1^+^ NK cells served as controls for the effect of educated KIR3DL1^+^ NK cells on HIV disease outcomes. Among the combined *KIR/HLA* genotypes associated with slow HIV disease progression were co-carriage of the *KIR3DL1-null* allele KIR3DL1*004 with *HLA-Bw4*, co-carriage of alleles encoding KIR3DL1-high and KIR3DL1-low allotypes with HLA-B*57 and HLA-B*27 and alleles encoding KIR3DS1 and Bw4*80I [67,68]. These studies implicated a role for NK cells in the natural history of HIV infection. These findings also raised the possibility that genetic combinations encoding certain *KIR/HLA* combinations may have accounted for the higher NK cell activity seen in IDU HESN individuals than PLWH enrolled in the Vietnamese IDU cohort. This could be the case if the enhanced NK cell activity observed in IDU HESN persons compared to IDU PLWHs pre-seroconversion was due to the contribution of these combined genotypes to the potency of NK cell education, which would have been directly related to levels of NK cell functionality in response to HIV-infected cells present in parenteral exposures.

As innate immune cells, NK cells are primed to respond to HIV-infected cells by eliciting anti-viral activity at the earliest stages of HIV infection in a manner that may prevent the establishment of HIV infection [18,24,28,69]. This prompted an investigation of whether *KIR/HLA* combinations associated with slower rates of HIV disease progression were also associated with prevention from HIV infection. This question was addressed by comparing the proportion of longitudinally followed IDU HESN persons and recently HIV-infected individuals carrying various *KIR/HLA* combinations associated with slower time to AIDS [70,71,72,73]. Carriage of the *KIR3DL1-high*+HLA-B*57 genotype combination, which had the most potent effect on viral load control and slow time to AIDS in PLWH was significantly more frequent among IDU HESNs than in recently infected PLWH [67,70]. These observations implicated a role for potently educated NK cells in protection from HIV infection.

While the combination of at least one copy of *KIR3DS1* with HLA Bw4*80I was associated with slower time to AIDS and viral load control, it was not associated with protection from HIV infection [68,71]. However, the carriage of the *KIR3DS1* homozygous genotype was linked to a reduced risk of HIV infection [71]. This genotype was also associated with slower time to HIV seroconversion in a highly HIV-exposed longitudinally followed IDU HESN cohort [74]. The proportion of *KIR3DL1/S1* heterozygotes and *KIR3DL1* homozygotes did not differ between IDU HESNs and recently infected PLWH. Co-carriage of *KIR3DS1* with HLA-Bw4*80I did not modify the effect on protection from infection conferred by the *KIR3DS1* homozygous genotype [71]. Not all the *KIR3DL1/S1/HLA* combined genotypes associated with a slower time to AIDS were also associated with protection from infection. This could be due to the size of the IDU HESN and recently infected PLWH populations studies being too small to provide sufficient power to observe significant between-group differences in the frequency of all the KIR3DL1/S1/HLA combinations that were associated with slower time to AIDS in longitudinally followed PLWH. An alternative possibility is that the mechanisms of protection from HIV infection differ from those related to rate of HIV disease progression in PLWH.

## 5. The Contribution of NK Cell Education to Function

When HLA-null cells stimulate NK cells, negative signaling through inhibitory NKRs is interrupted while signals through activating NKRs persist [75]. In this scenario, NK cells are activated in accordance with the potency with which they were educated, revealing their functional potential. HLA-null cell stimulation of NK cells from HIV uninfected carriers of the *KIR3DL1-high*+B*57 genotype combination activated a higher frequency of NK cells to secrete interferon-γ (IFN-γ), the chemokine CCL4, and externalize CD107a, a marker for NK cell degranulation than NK cells from carriers of alleles encoding other KIR3DL1/HLA receptor/ligand pairs [76]. HLA-null cells also stimulated a higher frequency of NK cells from KIR3DL1*004+*Bw4* carriers than *Bw6* homozygotes to secrete IFN-γ and CCL4 and externalize CD107a, though this combination was not more frequent among IDU HESN individuals than in recently infected PLWH [77].

**Figure 2:**
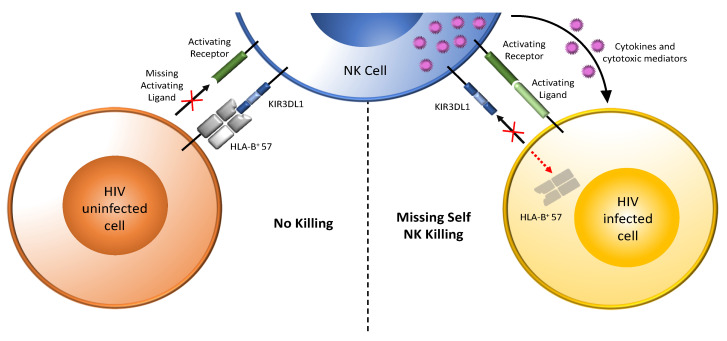
Target recognition, tolerance, and missing-self recognition. NK cells recognize and kill their targets through the integration of signals received from inhibitory and activating receptors. They can discriminate between healthy cells (tolerance) and eliminate transformed or virally infected targets (killing). As shown to the left of the vertical dotted line that separates the figure into two parts, NK-cell tolerance depends on the interaction of several major histocompatibility complex (MHC) class I ligands (either classical, HLA-A, -B, or -C, or nonclassical, HLA-E) expressed by heathy cells with their inhibitory KIR or NKG2A receptors with minimal activation signals. Shown here as an example is expression of the MHC class I antigen HLA-B*57 on healthy cells engaging the inhibitory receptor KIR3DL1 on NK cells, transducing inhibitory signals that maintain NK cells in a resting state. HIV uninfected cells express few ligands for activating receptors. On the right, HIV-infected cells downmodulated HLA, abrogating inhibitory signals through KIR3DL1. HIV-infected cells also upregulate ligands for activating NK cell receptors. Loss of inhibitory signaling (as indicated by the “+” symbol over the ligand for inhibitory KIR3DL1) and gain of activating signaling activates NK cells to release cytokines and cytotoxic mediators, which kill HIV-infected cells. Dark green bar on the NK cell = activating NK cell receptor; light green bar on HIV infected cell (right panel) = ligand for an activating receptor (this bar in missing on HIV uninfected cells in left hand panel as indicated by the “+” symbol); blue three domain structure on the NK cell = inhibitory KIR3DL1 receptor; grey 4 domain structure on HIV uninfected cells (left-hand panel) = HLA-B*57, a ligand for inhibitory receptor KIR3DL1 (this HLA antigen is missing on the HIV infected cell in left hand panel as indicated by the “+” symbol); purple dots = cytokines and cytotoxic mediators.

HIV-infected CD4 cells downmodulate cell surface HLA expression and upregulate the expression of ligands for activating NKRs, which together activate NK cells (Figure 2) [21,22,23,25,69]. NK cell activation by autologous HIV-infected cells was observed by gating on NK cells expressing a single inhibitory KIR by flow cytometry. The frequency of single positive KIR3DL1^+^ NK cells secreting IFN-γ and CCL4 and externalizing CD107a, all of which have anti-HIV activity, was higher in educated than uneducated, KIR3DL1^+^ NK cells from *Bw6* homozygotes responding to autologous HIV-infected CD4 cell stimulation [69]. Expression levels of both the KIR3DL1 receptor and its HLA-B ligand and their binding affinity influenced single positive KIR3DL1^+^ NK cell education potency and responsiveness to autologous HIV-infected CD4 cells [35,69]. For example, NK cells from *KIR3DL1-high*+HLA-B*57 carriers had a superior ability to inhibit HIV replication in autologous infected CD4 cells compared to carriers of other *KIR/HLA* combinations and *Bw6* homozygotes [28]. The inhibition of HIV replication was mediated, at least in part, by soluble CCL3, CCL4, and CCL5 chemokines secreted by activated NK cells because blocking all three of these chemokines restored HIV replication [28]. These three chemokines bind CCR5, the co-receptor for HIV entry, thus blocking the infection of new HIV susceptible CD4 target cells [27].

IDU HESN individuals, compared to recently infected PLWH, were more likely to carry two copies of *KIR3DS1* and one or two copies of a telomeric KIR t*B01* motif and were less likely to carry a full length KIR2DS4*001-like allele [78]. A study comparing 25 IDU HESN persons with 19 IDU PLWH and 26 HIV uninfected individuals from the Vietnamese IDU cohort mentioned above found that HESN persons had KIR expression profiles encoding higher ratios of *KIR3DS1*:*KIR3DL1* transcripts [79]. A higher prevalence of *KIR3DS1* or lower frequency of *KIR3DL1* alleles in HESN individuals than in HIV-susceptible subjects has been reported in several studies, and this is often in the absence of an association with *HLA-Bw4* alleles [71,74,79,80,81]. Overall, these studies support the interpretation that HIV resistance may be due to NK cells that are more easily activated, which is consistent with carriage of a group *B KIR* haplotype in which larger numbers of genes encoding activating KIRs are present.

*KIR3DS1* is found on the telomeric KIR *tB01* motif in linkage disequilibrium with *KIR2DL5A*, *KIR2DS1,* and *KIR2DS5* genes (Figure 1). Although these genes are in linkage disequilibrium, their gene products are expressed on NK cells in a stochastic fashion, allowing investigation of the functional potential of NK cells expressing various combinations of the KIRs encoded by these genes following stimulation with the HLA null cell line 721.221. NK cells that responded with the highest frequency of functional cells were KIR3DS1^+^. Co-expression of KIR2DL5, KIR2DS1, or KIR2DS5 did not modulate the frequency of responding KIR3DS1^+^ NK cells nor did KIR2DL5^+^, KIR2DS1^+^, and KIR2DS5^+^ NK cells respond to this stimulus any better than their KIR2DL5^−^, KIR2DS1^−^, or KIR2DS5^−^ counterparts. Thus, of the KIR receptors encoded by genes present within the *tB01* motif, KIR3DS1 contributes most to NK cell responsiveness to 721.221 HLA null cells [78].

Attempts to find ligands for KIR3DS1 among HLA-A, -B and -C allotypes, including HLA-Bw4 and HLA-Bw4*80I allotypes, using beads expressing single HLA antigens failed [82,83]. KIR3DS1 did interact with the HLA-Bw4*80I antigen, HLA-B*57, when certain HIV-derived peptides were present [84]. If the interaction of KIR3DS1 with HLA-B*57 was contributing to NK cell education, this interaction should tune down KIR3DS1^+^ NK cell responsiveness to 721.221 stimulation as occurs when the activating NKR KIR2DS1 interacts with HLA-C2 group antigens [85,86]. KIR3DS1 binds the open conformation of HLA-F. KIR3DS1^+^ NK cells are activated by their interaction with HLA-F, expressed on 721.221 cells [82,87,88,89]. NK cells, stimulated with the HLA-F expressing 721.221 cells and autologous HIV-infected CD4 cells, stimulated a higher frequency of NK cells to produce IFN-γ, CCL4, and CD107a than control stimuli [24,82,87]. Blocking this interaction with either antibodies to HLA-F or KIR3DS1-Fc chimeric proteins significantly reduced KIR3DS1^+^ NK cell activation. Since HLA-F is usually intracellular in resting cells, it would not be expected to contribute to NK cell education in vivo and tune down the responsiveness of KIR3DS1^+^ NK cells to HLA-F expressing cells [89]. However, HLA-F is cell surface expressed on activated cells, including HIV-infected CD4^+^ T cells [24,82,89]. The interaction of KIR3DS1 on NK cells with HLA-F on HIV-infected cells may explain why KIR3DS1^+^ NK cells are superior to KIR3DL1^+^ NK cells in suppressing HIV replication [82,90]. This interaction may provide a mechanistic explanation for the reduced risk of HIV infection observed in IDU HESN *KIR3DS1* homozygotes, i.e., the rapid activation of these NK cells by interactions with HLA-F-expressing HIV-infected cells to induce anti-HIV functions.

NK cells from donors with no *KIR2DS4* gene, or who carry only truncated KIR2DS4*003-like alleles do not express the KIR2DS4 receptor on their cell surface, while NK cells from carriers of the full length KIR2DS4*001-like alleles express this receptor on a subset of their NK cells. KIR2DS4 was associated with poor outcome in the context of HIV infection such as higher viral load, lower CD4 counts and HIV transmission in HIV discordant couples [91,92]. This observation was confirmed by Olvera et al. in another group of PLWH from Lima, Peru and may have depended on the co-expression of HLA-Cw4, a presumed ligand for this receptor [93]. Expressed full length KIR2DS4 is linked with reduced resistance to HIV infection [78]. The mechanism underlying this finding is not understood. KIR2DS4 is an activating KIR that is the product of gene conversion between *KIR2DL* genes and the *KIR3DL2* gene that has led to a reduced ability to recognize HLA-C ligands characteristic of KIR2DL receptors and an increased ability to recognize HLA-A*11:02 and HLA-A*03 ligands, the presumed ligands for KIR3DL2 [94]. Further investigation is needed to understand whether, and if so how, KIR2DS4 expression is associated with negative outcomes in the context of HIV infection and HIV exposure. The possibility that the impact of expression of KIR2DS4 on poor HIV outcomes is due to other genes in linkage disequilibrium with *KIR2DS4* has not been excluded.

Together, these observations implicate a role for NK cell activation in protection from HIV infection. This can occur through the potent education of NK cells that become activated upon loss of inhibition mediated by the downmodulation of ligands for inhibitory NKR receptors on autologous HIV-infected CD4^+^ T cells combined with the upregulation of ligands for activating receptors on these cells. KIR3DS1^+^ NK cells can also be activated by the upregulation of HLA-F and other ligands for activating receptor on HIV-infected CD4 cells [21,24]. Once activated, these NK cells rapidly elicit functions that can not only control HIV but may also prevent infection of new target cells.

## 6. Alloreactive NK Cells in Protection from Sexual HIV Transmission

Haploidentical hematopoietic stem cell transplants (HSCT) are used to treat acute myeloid leukemia and acute lymphoid leukemia. T cells are depleted from haploidentical HSCTs to reduce graft versus host disease mediated by T cells activated by allogeneic cells. NK cells in these haploidentical HSCTs mediate a graft versus leukemia effect if alloreactive educated NK cells respond to absence of ligands for inhibitory KIR on leukemia cells, leading to a reduced risk of leukemia relapse [95,96,97]. This finding was the basis for investigating whether alloreactive NK cells had a protective effect in the context of sexual HIV transmission. The effect needed to be investigated in sexual partners where the *KIR/HLA* combinations of both partners was available.

While still debated, studies infecting rhesus macaques with simian immunodeficiency virus favor a model in which simian immunodeficiency virus infected CD4 cells rather than free virions are the predominant mechanism for transmission [98,99,100,101]. By analogy, new HIV infections arising from sexual exposures occur, at least in part, from HIV-infected cells present in genital secretions [101]. Thus, for an immune response to be effective at preventing infection, it needs to be able to target incoming HIV-infected cells early in infection before resting CD4 cell HIV reservoirs are established [15,16]. NK cells can target HIV-infected CD4 cells and are present as resident cells throughout the vaginal, uterine, and gut mucosa [102]. NK cells are rapidly activated by allogeneic HIV-infected CD4 cells. The HIV Nef and Vpu driven downmodulation of HLA-A, -B, and -C antigens from the surface of HIV-infected cells can further contribute the activation of the recipient’s NK cell and the susceptibility of incoming allogenic cells to NK cell-mediated control [23,25,69,103,104,105].

**Figure 3:**
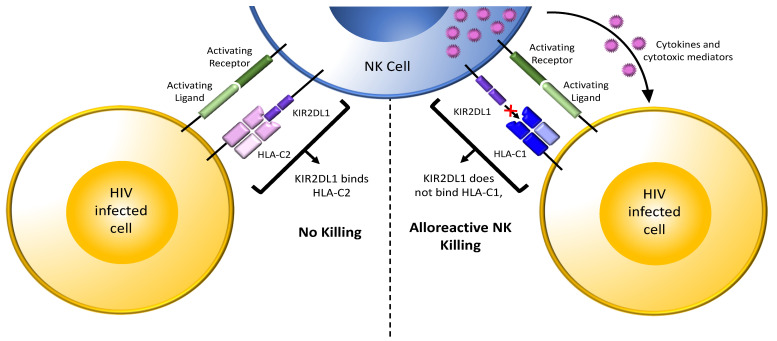
NK cell responses to allogenic cells. Shown in the top middle is an NK cell from an HIV uninfected person expressing the inhibitory receptor/ligand combination KIR2DL1/HLA-C2. The interaction of KIR2DL1 with HLA-C2 during development educates these NK cells. When educated KIR2DL1^+^ NK cells in a recipient partner encounter incoming HIV-infected CD4 cells from a transmitting partner who is HLA-C2^+^ (bottom left-hand cell, matched combination), the interaction of HLA-C2 on infected cells with the KIR2DL1 inhibitory receptor on recipient partner NK cells inhibits NK cell activation despite HIV-infected cells having ligands for activating receptors on NK cells. In this scenario, cytolysis of the HIV-infected cells is inhibited, increasing the chance of HIV transmission. On the right, educated KIR2DL1^+^ NK cells in a recipient partner encounter incoming HIV-infected CD4 cells from a transmitting partner who is HLA-C1^+^ (bottom right-hand cell, unmatched combination). Absence of inhibitory signaling through KIR2DL1 together with the presence of activating signals from activating receptors interacting with their ligands on HIV-infected cells activates NK cells. Activated NK cells release cytokines and cytotoxic mediators, which suppress and/or kill incoming HIV-infected cells and reduce the chance of HIV transmission. Light green bar = ligand for an activating receptor; dark green bar on the NK cell = activating NK cell receptor; purple 2 domain structure on the NK cell = inhibitory NK cell receptor KIR2DL2; light pink 4 domain structure on the HIV infected cell (left) = HLA-C2, a ligand for inhibitory receptor KIR2DL1; blue 4 domain structure on the HIV infected cell (right) = HLA-C1, unable to bind inhibitory receptor KIR2DL1; purple dots = cytokines and cytotoxic mediators; “+” symbol between KIR2DL1 and HLA-C1 on the right indicated the absence of an interaction between this receptor and ligand pair.

Several reports showed that HLA mismatches between transmission pairs decreased the risk of perinatal and sexual HIV transmission [106,107,108,109,110]. *KIR/HLA* genetic variation, particularly combinations having an activating *KIR/HLA* profile such as activating KIRs, a group *B KIR* haplotype, or inhibitory KIRs in the absence of their ligands were associated with protection from infection in HESN individuals [71,74,78,79,80,91,111]. Jennes et al. investigated the role of NK cells in HIV resistance in the seronegative partners of serodiscordant couples and susceptibility to HIV in seroconcordant couples [105]. They hypothesized that, in certain contexts, NK cells were activated by allogeneic HIV-infected cells to kill infected cells originating from their sexual partners [105]. They found that HIV transmission in heterosexual couples depended on the presence of NK cell educating *KIR/HLA* combinations in the recipient partner. If the transmitting partner expressed MHC class I allotypes that were not recognized by the recipient partner’s educated NK cells (i.e., were mismatched) they were less likely to seroconvert than if the transmitting partner expressed MHC class I antigens recognized by the recipient partner’s NK cells (i.e., were matched) (Figure 3).

Presumably, educated NK cells in the recipient partner were more likely to be activated by incoming allogeneic HIV-infected cells to reduce the risk of HIV transmission in the mismatched but not in the matched situation [105]. This idea was tested in vitro by co-culturing NK cells with allogeneic HIV-infected CD4 cells. These experiments confirmed that NK cells killed a higher frequency of missing self than matched HIV-infected cells. Later experiments further refined these observations by showing that both the frequency of lysed HIV-infected CD4 cells and the frequency and intensity of CD107a externalization by NK cells activated by allogeneic HIV-infected CD4 cells were higher in mismatched than in matched combinations [112]. NK cells from subjects carrying haplotype *B KIR* genotypes with activating KIRs contributed to greater levels of NK cell activation than when no activating KIRs were present [112]. HIV-infected cells also upregulate several ligands for activating NK cell receptors, which could further contribute to activating NK cell responses to HIV-infected cells [21]. MHC class I downmodulation by HIV Nef and Vpu may further contribute to the vulnerability of the incoming infected CD4 cells from transmitting partners who are PLWH to recipient NK cell responses. Together, these findings confirm previous observations that activating NK cell genotypes and inhibitory KIRs in the absence of their HLA ligands were associated with HIV resistance and suggest mechanisms that may underlie these observations [71,78,80,111,113].

It is notable that HIV serodiscordant couples were more likely to be composed of an uninfected recipient partner with an NK cell educating *KIR/HLA* combination such as *KIR2DL1*+*HLA-C2* and a PLWH partner who was homozygous for C1 than seroconcordant couples (Figure 3) [105]. The contribution of *KIR2DL*+*HLA-C KIR/HLA* combinations to protection from HIV infection by the heterosexual transmission route appears to be greater than that of *KIR3DL1/S1*+*Bw4* combinations [112]. The implication of *KIR2DL*+*HLA-C* combinations in protection from sexual HIV transmission complements the observation made for *KIR3DL1*+HLA-B*57 and KIR3DS1 in protection from parenteral HIV infection in IDU HESN individuals. More work is needed to understand whether *KIR/HLA* combinations contribute differentially to protection from infection according to the route of HIV transmission.

## 7. Mechanisms of Protection from HIV Infection

NK cells dependent mechanisms of host resistance to HIV infections described above have focused on the role of activated NK cells. Others have also implicated NK cell activation in in protection from HIV infection. Tomescu et al., working with a cohort of IDU HESN persons, found persistently higher frequencies of activated NK cells and monocyte-derived dendritic cells among HESN individuals sharing needles than among low-risk non-sharing IDU or non-drug using controls [114]. The enhanced activation of these cell types in IDU HESN did not appear to be due to the injection drugs used but rather due to other factors related to drug use such as exposure to pathogens or allogeneic cells from injection partners. A proteomic analysis of NK cells from IDU HESN individuals and healthy control donors found that the protein S100A14 was upregulated in the former group. A100A14 is a member of the S100 family that are involved in many cellular processes. Although these proteins have been studied in the context of cancer, little is known about their role in the setting of NK cells and HIV. This molecule activated NK cells in a monocyte-dependent manner that required NK cell monocyte cell contact. S100A14 activated monocytes by signaling through toll-like receptor 4 to secrete TNF-α. These findings suggest that S100A14 is involved in NK cells monocyte crosstalk that promoted NK cell activation in IDU HESN [115].

It should be noted, however, that there is unlikely to be a unified protective mechanism for protection from HIV in HESN, as both immune quiescence [116,117] and immune activation [118,119,120,121,122] have been identified depending on the route of exposure. Studies conducted in a highly HIV-exposed commercial sex worker (CSW) cohort followed in Nairobi, Kenya identified immune quiescence as a mechanism for resistance to HIV infection. A subset of these CSWs remained uninfected despite 7 or more years of HIV exposure [123]. These resistant CSW HESNs are distinguished from their HIV-susceptible counterparts by maintaining an immune profile characterized by quiescence [116]. As HIV infects and replicates preferentially in activated CD4^+^ T cells, the ability of HIV-susceptible CD4^+^ cells at the portals of entry to remain quiescent despite HIV exposure may preclude HIV replication in these cells and reduce the risk of establishing a productive infection [116]. A role of NK cells in immune quiescence has not been described.

## 8. Adaptive NK Cells in Protection from HIV Infection

Human cytomegalovirus (HCMV) infection expands a population of NK cells with adaptive-like features [124]. These adaptive NK (adapNK) cells express the activating receptor, NKG2C, which belongs to the C-type lectin family [125]. The NKG2C activating receptor, like its inhibitory counterpart, NKG2A, is expressed as a heterodimer with CD94 [126]. The ligand for NKG2C and NKG2A is HLA-E, a nonclassical MHC class Ib antigen stabilized by peptides derived from leader sequence of classical MHC class I antigens, the nonclassical MHC Ib HLA-G antigen or epitopes from the HCMV-encoded viral protein UL40 [39,127,128]. The interaction of NKG2C with its ligand transmits signals that activate cells expressing this receptor [39,129]. AdapNK cells undergo DNA methylation-dependent epigenetic modifications, which distinguish them from conventional NK cells and influence their functionality [130,131].

Some individuals do not express cell surface NKG2C due to a homozygous deletion of approximately 16 kb that includes the *nkg2c* gene encoding NKG2C [132,133]. In several Caucasian populations, a Japanese cohort, and a Tanzanian cohort, the frequency of the *NKG2C* deletion allele is close to 20% with a homozygous deletion genotype frequency of approximately 4% [133,134,135,136]. Several studies have questioned whether NKG2C^+^ cells play a role in protection from HIV infection or in rate of HIV disease progression in PLWH. Thomas et al. compared the distribution of NKG2C genotypes (*NKG2C^+/+^*, *NKG2C^+/−^* and *NKG2C^−/−^*) in 433 PLWH and 280 uninfected individuals with no history of HIV exposure. They reported that that carriage of at least one *NKG2C^-^* variant (i.e., the NKG2C^+/*−*^ and NKG2C*^−^*^/*−*^ genotypes) was associated with a higher risk of HIV infection [137]. In a separate study comparing 434 PLWH and 157 HESN persons there were no between-group differences for the frequency of either the *NKG2C^+/+^* or the combined *NKG2C^+/−^* and *NKG2C^−/−^* genotypes [138]. However, the frequency of the *NKG2C^−/−^* genotype was higher among PLWH than HESN individuals. None of the 157 HESN subjects tested carried the NKG2C*^−^*^/*−*^ genotype, which was present in 11 of 434 (2.53%) PLWH. Of note, the distribution of *NKG2C* genotypes did not differ in the PLWH and HESN sub-populations who were sexually exposed to HIV, while the *NKG2C^−/−^* genotype was more frequent in parenterally exposed PLWH than in HESN individuals. These findings suggest that the *NKG2C^−/−^* genotype is associated with a higher risk of HIV infection by parenteral, but not by sexual exposure [138].

In Thomas at al., the control population was not HIV-exposed and thus was at a low risk for HIV infection, while in the study by Alsulami et al. the HIV seronegative population were HESN persons at high risk for HIV exposure and more likely to have some level of resistance to HIV infection. Thus, the inclusion of HESN participants allowed for a more direct exploration of whether *NKG2C* genotypes were associated with HIV susceptibility.

What accounts for the frequency of the *NKG2C^−/−^* genotype not differing significantly between sexually exposed PLWH and HESN persons is unclear. To better understand this phenomenon, more information is needed regarding factors that influence the per act risk of HIV transmission by different routes and the NK cell, and specifically the NKG2C^+^ NK cell, landscape at mucosal portals of HIV entry. Additionally, factors such as treatment status and HCMV serostatus need to be accounted for.

## 9. Can Vaccines Induce NK Cell Responses That Prevent HIV Infection?

The RV144 HIV vaccine trial induced modest, though significant, protection against HIV infection [139]. Correlates of protection analyses found that vaccine-induced antibodies able to bind HIV Envelope structures and support NK cell-mediated antibody-dependent cellular cytotoxicity (ADCC) were associated with protection from HIV infection [140]. A vaccine regimen similar to the one used in the RV144 vaccine trial, designed for use in South Africa, where HIV clade C predominates, failed to show efficacy in preventing HIV infection [141]. This was despite it inducing immune responses analogous to those seen in the RV144 trial. Nevertheless, the RV144 trial offers an example of a role for NK cells in protection from HIV infection. Presumably, the HIV Envelope-specific IgG antibodies induced by this vaccine were able to bridge HIV-infected cells expressing cell surface HIV Envelope with CD16 on NK cells. Engagement and crosslinking of CD16 would have resulted in NK cell activation and the lysis of incoming HIV-infected cells by ADCC. This finding provides an impetus for investigating strategies that can improve the efficacy of vaccine-induced immune responses that protect from infection.

Early in an immune response, NK cells secrete cytokines and chemokines such as IFN-γ, TNF-α, CCL3, CCL4, CCL5, and granulocyte-macrophage colony-stimulating factor [142]. These secreted factors contribute to recruiting and activating antigen-presenting cells such as dendritic cells, which in turn collaborate with T and B cells to induce adaptive immunity [143]. IFN-γ derived from NK cells promotes type 1 T helper cell differentiation and stimulates isotype class switching in B cells [144,145]. Designing vaccines that harness NK cell activity has the potential to improve the induction of adaptive immune responses able to collaborate with NK cells to prevent HIV infection [146]. The incorporation of novel adjuvants into vaccine formulations may induce NK cell activities that increase adaptive immune responses [147,148,149,150].

One goal would be to design vaccines that activate NK cells to promote B cells to produce antibodies able to bind to Envelope on HIV-infected cells that are ADCC competent. The presence of such antibodies able to opsonize incoming HIV-infected cells would have the potential to lead to their lysis through ADCC.

Human cytomegalovirus (HCMV) infection drives the expansion of adaptive-like NK cells expressing NKG2C and/or that are negative for FcRγ and other signaling molecules. These adaptive-like NK cells have epigenetic changes that confer antibody dependent effector functions such as ADCC activity that is superior to that seen in conventional NK cells [124,130,131,151,152]. Vaccine strategies aimed at inducing NKG2C^+^ NK cells in the absence of CMV infection are being investigated [153].

However, NK cells can also negatively regulate immune responses through their cytolytic activity. NK cell-mediated cytolysis can reduce vaccine antigen persistence, eradicate responding T cells, suppress the establishment of adaptive memory, and block the evolution of antibody responses [143,154,155]. The dichotomous activity of NK cells adds complexity to targeting these cells through vaccination. Future investigations will need to consider this issue when designing vaccines targeting NK cells.

## 10. Concluding Remarks and Future Directions

HESN individuals can be found among IDUs, HIV serodiscordant couples, CSWs, and perinatally exposed infants. Studying HESN persons may provide clues to mechanisms that prevent HIV infection. There is evidence of a role of NK cells in protection from HIV infection through parenteral and sexual routes of exposure.

In terms of parenteral exposure, information on the transmitting partners is rarely available. Thus, epidemiological studies have relied on whether certain *KIR/HLA* combinations are associated with protection from HIV infection. Those that are, tend to be combinations that favor NK cell education for potent responses to autologous HIV-infected cells in the case of the *KIR3DL1*+HLA-B*57 combination and NK cell activation through ligation of the KIR3DS1 activating receptor on NK cells by ligands on HIV-infected cells as in the case of *KIR3DS1*+*HLA-F* combinations. In both these cases, NK cells from carriers of both these *KIR/HLA* combinations are induced to secrete higher levels of cytokines and chemokines and externalize higher levels of CD107a when co-cultured with autologous HIV-infected cells that all have anti-HIV activity.

In HIV serodiscordant and seroconcordant couples, the identity of both the recipient and transmitting partners is often available. By typing recipient partners for KIR and HLA and transmitting partners for HLA, we have learned that HIV discordance is more likely when the recipient partner has educated NK cells that cannot recognize HLA on the transmitting partner’s cells. In this scenario, recipient NK cells can respond to incoming allogeneic HIV-infected cells in a manner that reduces the risk of HIV infection. Lack of inhibition together with the induction of ligands for activating NK receptors on infected cells can favor NK cell activation in HESN recipient partners in this unmatched allogeneic context [105,112].

Several questions remain to be addressed. The role of the route of HIV exposure in determining whether *KIR3DL1/S1* and *HLA-Bw4/HLA-F* combinations are more potent in parenteral exposures while *KIR2DL/HLA-C* combinations are more effective at preventing HIV infection by sexual routes of exposure is unclear. We still have much to learn regarding the anatomy of NK (or ILC1) cell distribution at portals of HIV entry through sexual transmission. The contribution of immune quiescence versus NK cell activation to protection from HIV infection require further investigation.

NK cells not only respond directly to autologous and allogeneic cells’ missing ligands for inhibitory receptors but also mediate potent ADCC. To take advantage of this type of NK cell function, antibodies are required. It may be possible to design novel next-generation vaccines that harness the potential of NK cells to induce B cells to secrete antibodies that recognize the Envelope of HIV-infected cells rather than uninfected bystander cells [156,157,158]. The presence of such antibodies able to opsonize incoming HIV-infected cells would be effective targets for NK cell-mediated ADCC activity occurring soon enough after exposure to prevent the establishment of infection. Novel vaccine adjuvants may help induce NK cells to promote protective rather than detrimental NK cell functions. Inducing adaptive NK cells through vaccination may generate NK cells with superior ADCC activity that plays a role in preventing HIV infection.

## Figures and Tables

**Table 1:** Receptor-ligand pairs contributing to NK cell education.

HLA	Receptor
HLA-C1 (Asn 77)	KIR2DL2 (major), KIR2DL3
HLA-C2 (Lys 80)	KIR2DL1, KIR2DL2 (minor), KIR2DS1
HLA-Bw4	KIR3DL1
HLA-F (open conformation)	KIR3DS1
HLA-A*03, HLA-A*11	KIR3DL2
HLA-E presenting epitopes	NKG2A
from the HLA leader sequences	

## Data Availability

Not applicable.
